# Study of *Akkermansia muciniphila* Effect on the Gut Microbiome of Mice Under LPS-Induced Systemic Inflammation

**DOI:** 10.1155/ijin/8695182

**Published:** 2025-11-29

**Authors:** Mikhail Yu. Syromyatnikov, Inna Yu. Burakova, Yuliya D. Smirnova, Polina D. Morozova, Svetlana V. Pogorelova, Egor A. Chirkin, Anna A. Tolkacheva

**Affiliations:** ^1^Laboratory of Metagenomics and Food Biotechnology, Voronezh State University of Engineering Technologies, Voronezh, Russia; ^2^Department of Genetics, Cytology and Bioengineering, Voronezh State University, Voronezh, Russia

## Abstract

Probiotics are strains of living bacteria and yeast that play an important role in regulating the gut microbiota and enhancing host immunity. In the last decade, the bacterial species *Akkermansia muciniphila* has attracted great interest due to its possible probiotic properties, which play an important role in human health. However, the mechanisms of action of *A. muciniphila* are still poorly understood. The effect of the *A. muciniphila* on the intestinal microbiome of model animals with systemic inflammation induced by lipopolysaccharide (LPS) is unexplored. This study aims to investigate the impact of *A. muciniphila* on the microbiological composition of the mouse gut under LPS-induced systemic inflammation using high-throughput sequencing. The study used a new generation sequencing method aimed at genome-wide sequencing of microorganisms, which makes it possible to study changes in the composition of the microbiome at the bacterial species level, as well as to identify the genes of the metabolic pathways of intestinal bacteria in the studied mice. Our analysis revealed statistically significant differences across all studied groups, with a notable predominance of members from the families Muribaculaceae, Rikenellaceae, and Oscillospiraceae. Consumption of *A. muciniphila* increased the alpha diversity of gut bacteria (Shannon index) in the context of induced inflammation. Evaluation of the effect of LPS and *A. muciniphila* on metabolic pathways showed statistically significant differences for the pathways of synthesis and degradation of amino acids, transforming folic acid, and synthesis of sugars. Genetic analysis showed that the probiotic bacterium *A. muciniphila* reduced the degree of negative effects of LPS on the mouse gut microbiome under systemic inflammation.

## 1. Introduction

Probiotics are popular means of preventing and treating diseases of various etiologies, including diseases of the gastrointestinal tract [1], metabolic diseases [2], diseases of the nervous system [3], etc. Standard probiotics typically include lactobacilli, bifidobacteria, as well as bacteria of the genera *Streptococcus*, *Enterococcus*, *Bacillus*, and some others [4, 5]. The search for new probiotic bacteria is constantly underway. In the last two decades, the significant role of the anaerobic bacterium *Akkermansia muciniphila* in maintaining a healthy human gut microbiome has been revealed [6–8]. This became possible due to the widespread introduction of high-throughput sequencing in the study of the intestinal microbiota [9]. This bacterium has a number of features. A distinctive feature of *A. muciniphila* is that it uses mucin as a source of nitrogen and carbon for its vital activity [10]. This bacterium has been shown to have a positive effect on the course of diseases such as obesity [11], type 2 diabetes [12], irritable bowel syndrome [13], intestinal cancer [14], fatty hepatosis [15], etc. The action of this bacterium is manifested in improving the intestinal barrier function and modulating the immune system [16]. Recent studies have shown the ability of this bacterium to influence the gut–brain axis [17], thereby allowing probiotics based on this bacterium to be considered as therapies for neuropsychiatric diseases [18]. In general, this bacterium is currently considered as a new-generation probiotic [19, 20]. However, further research is needed to assess the safety and efficacy of these bacteria.

It is important to study the influence of *A. muciniphila* on the background of various inflammatory processes. The effect of *A. muciniphila* on the gut microbiome of model animals with systemic lipopolysaccharide (LPS)-induced inflammation is unexplored. There have been no studies on the effects of LPS injections on the bacterial gut species composition of animal models and the relative abundance of gut metabolic bacteria pathways. LPS is a component of the outer membrane of many Gram-negative bacteria [21]. This molecule is not only involved in infectious diseases but may also be involved in metabolic diseases such as obesity and diabetes [22]. New approaches are needed to neutralize the mechanisms of the pathogenic effect of LPS. Modulation of the gut microbiota may be one of these mechanisms. Understanding the pathways of the new-generation probiotic bacterium *A. muciniphila* against systemic inflammation will allow to develop new approaches in the treatment of diseases involving LPS molecules in the future. The aim of this work was to evaluate the effect of *A. muciniphila* on the gut microbiome in induced systemic inflammation caused by LPS administration using metagenomic sequencing to evaluate both the bacterial composition and the metabolic pathways of bacteria.

## 2. Materials and Methods

### 2.1. Design of study

Males of the *Mus musculus* C57BL/6 line from the Andreevka nursery (Moscow region, Russia) were used as a model organism. Mice were kept according to the guidelines for the care and maintenance of laboratory animals [23]. All the studied mice received a portion of food at a rate of 5 g/mouse. The study used the bacterium *A. muciniphila VSUET1AM* from the Museum of Microbiological Cultures of Voronezh State University of Engineering Technologies.

At the beginning of the study, the mice were divided into 4 groups:

1. Control group, “Control group” (*n* = 6);
2. The control group, which was injected with LPS, “LPS group” (*n* = 6);
3. A group of mice that consumed 1∗10^5^ cells of *A. muciniphila* bacteria through a gastric tube, but did not receive injections of LPS “Akkermansia group” (*n* = 7);
4. A group of mice that consumed 1∗10^5^ cells of *A. muciniphila* bacteria through a gastric tube and receive injections of LPS “Akkermansia + LPS group” (*n* = 7).

The drug “Pyrogenal” (Federal State Budgetary Institution “NITSEM named after N.F. Gamaleya” of the Ministry of Health of the Russian Federation) was chosen as the source of LPS 100 μg/mL. For injection, LPS was diluted 1:1 with saline solution and injected 200 μL intraperitoneally daily during the last week of the experiment. The duration of the experiment was 3 weeks.

Stool samples were collected from each mouse under study; each sample (about 100 mg) was collected in an Eppendorf tube using disinfected plastic equipment after defecation. The samples were immediately placed in a temperature-controlled freezer −40°C.

### 2.2. DNA Extraction

Total DNA was isolated from each stool sample using the MetaGen Kit (Syntol, Russia) following the manufacturer's protocol.

Then, the quality and quantity of total DNA obtained from the samples were evaluated. The amount of DNA was determined using a Nano-500 fluorimeter (Hangzhou Allsheng Instruments Co., Ltd. China) and the Equalbit 1*x* dsDNA HS Assay Kit (Vazyme, China). The quality of the obtained DNA was assessed using electrophoresis in 2% agarose gel.

### 2.3. cPAS Sequencing on the DNBSEQ-G50 Instrument

Preparation of sequencing libraries, as well as subsequent cPAS sequencing on the DNBSEQ-G50 instrument, was carried out in accordance with the protocol described previously [24].

### 2.4. Sequencing Data Processing and Statistical Analysis

The technical sequences were cut using flexbar. The isolation of human and host sequences from the samples was performed by comparing the metagenomic readings with the reference genomes of mice (GCF_000001635.27) and humans (GCF_000001405.40) by the Bowtie2 tool. In addition, taxonomic classification of the samples was performed using MetaPhlAn 4 (version 4.1.1) with standard databases (bacteria, eukaryotes, viruses). Microbial metabolic cascades were characterized using Human.

Statistical calculations were performed in the environment of *R* (Version 4.1.1). Alpha diversity was estimated using the Shannon index, and differences in it were determined using the nonparametric Mann–Whitney test. Beta diversity was analyzed using the Bray–Curtis difference metric.

To monitor differences in diversity between groups, the ADONIS function was used. Differences in species abundance were analyzed using the MaAsLin2 package (version 1.18.0) with a multivariate regression model. Statistically significant results were defined as *p* < 0.05. The results are presented as mean ± standard deviation (SD).

## 3. Results

**Figure 1:**
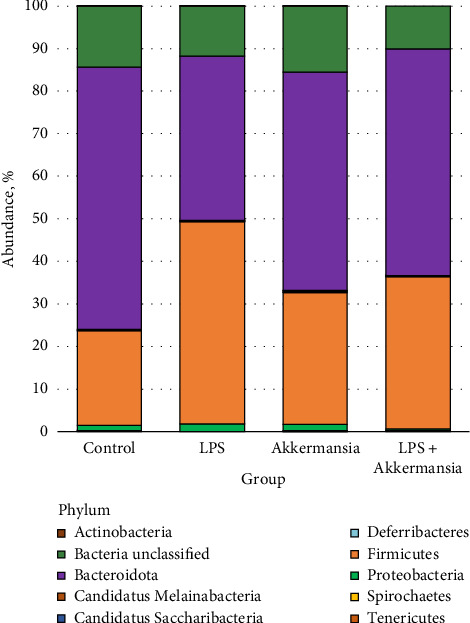
Taxonomic composition of fecal microbiota in the studied groups at the phylum level.

Analysis of the fecal microbiome of laboratory mice from the “Control group” and “LPS group,” “Akkermansia group,” “Akkermansia + LPS group” made it possible to identify 11 phyla, 84 classes, 85 orders, 100 families, 345 genera, and 436 species of bacteria (Figure 1). 326 bacterial species were identified as previously unclassified, indicating that the mouse gut microbiome is understudied.

**Figure 2:**
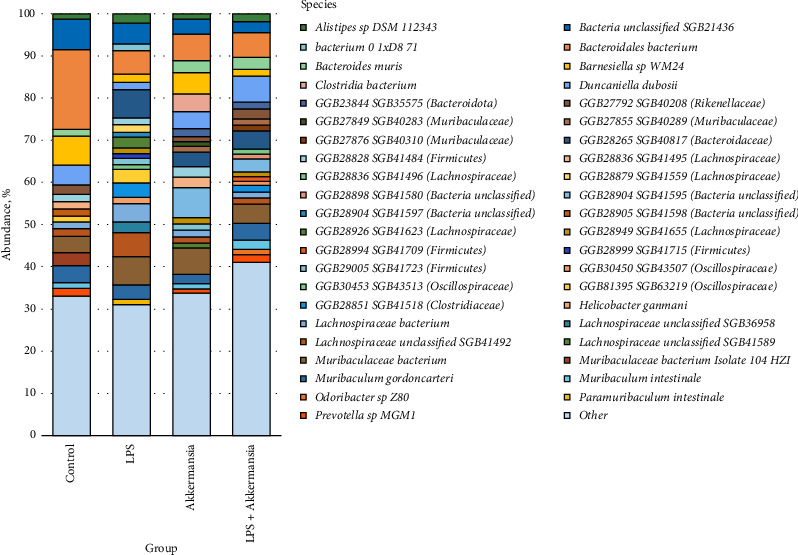
Bacterial species found in the microbiome of the studied groups.

A comparative analysis of the microbiome of the “Control group” and “LPS group,” “Akkermansia group,” and “Akkermansia + LPS group” was carried out. 44 of the most widespread species were identified, the number of which exceeded 1%; all other species were grouped as “Others” (Figure 2).

**Figure 3:**
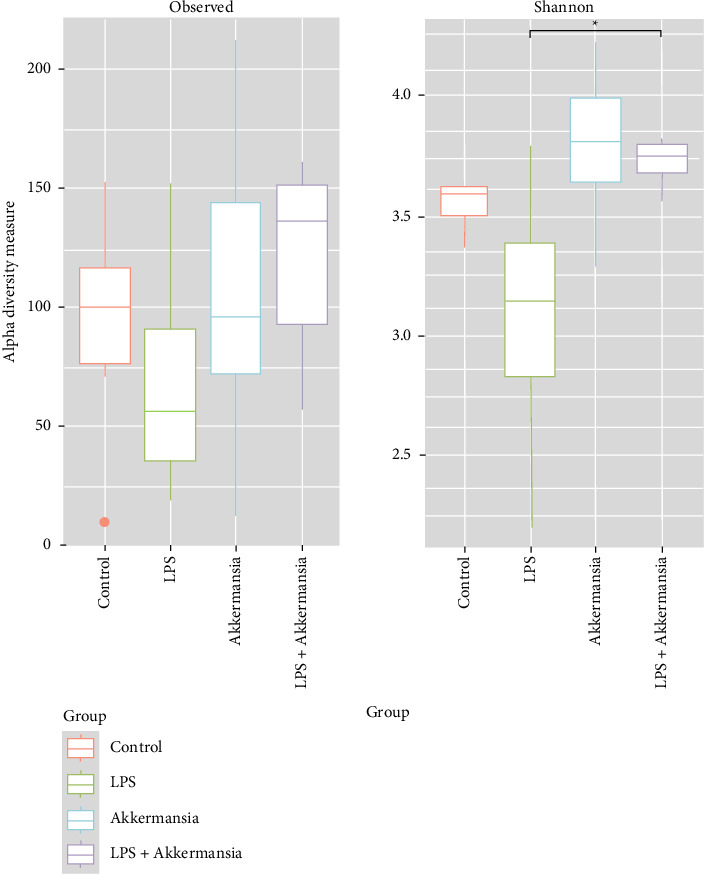
Alpha-diversity indicators of microbiota of the studied groups. ^∗^*p* ≤ 0.05.

The alpha diversity of the fecal microbiome was assessed using the Shannon index. In all study groups, its value exceeded 3, indicating a high level of diversity (Figure 3). Statistical analysis revealed a significant difference (*p* = 0.012) only between the “LPS group” and “Akkermansia + LPS group”.

**Figure 4:**
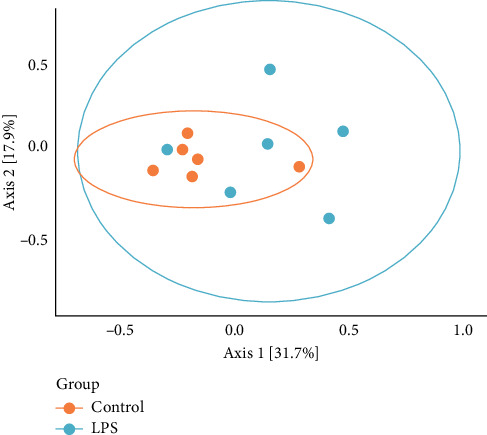
PCoA analysis of beta diversity based on the Bray–Curtis scale between the “control group” and “LPS group.”

Beta diversity analysis revealed clustering of samples between the study groups. The centroid of the control group was statistically significantly different from the centroid of the “LPS group,” *p* = 0.043 (Figure 4). No significant differences were found between the other groups.

**Figure 5:**
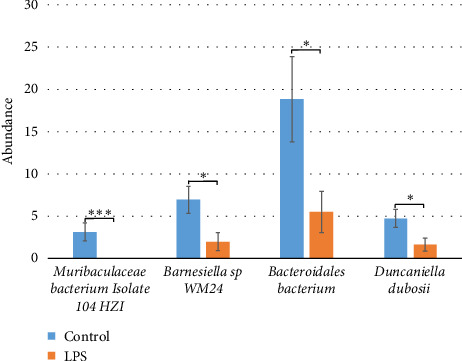
Differences in the composition of the fecal microbiome between the “control group” and “LPS group” for most abundant species (more than 1%). ^∗^*p* ≤ 0.05, ^∗∗^*p* ≤ 0.01, and ^∗∗∗^*p* ≤ 0.001.

**Figure 6:**
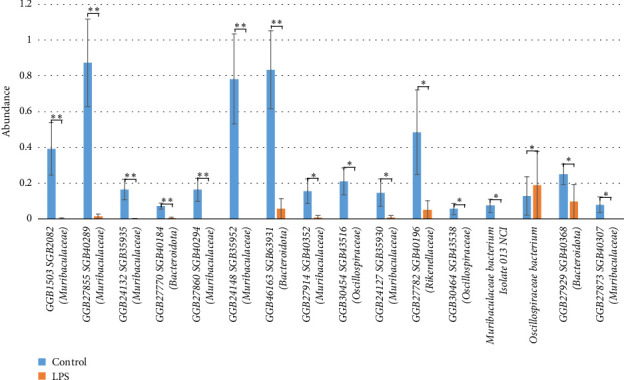
Statistically significant differences between the fecal microbiota of the “control group” and “LPS group” for minor species (less than 1%). ^∗^*p* ≤ 0.05 and ^∗∗^*p* ≤ 0.01.

Analysis of differential abundance revealed statistically significant intergroup differences at the species level between the “control group” and the “LPS group” (Figures 5 and 6). The control group had a higher abundance of *Muribaculaceae bacterium isolate 104 HZI* (3.13% ± 1.06 vs. 0, *p* = 2.1E-04), *GGB1503 SGB2082 (Muribaculaceae)* (0.39% ± 0.15 vs. 3.02E-03% ± 3.02E-03, *p* = 1.62E-03), *GGB27855 SGB40289 (Muribaculaceae)* (0.87% ± 0.24 vs. 0.01% ± 0.01, *p* = 1.15E-03), *GGB24132 SGB35935 (Muribaculaceae)* (0.16% ± 0.05 vs. 2.68E-03% ± 2.68E-03, *p* = 2.64E-03), *GGB27770 SGB40184 (Bacteroidota)* (0.07% ± 0.02 vs. 4.41E-03% ± 4.41E-03, *p* = 2.52E-03), *GGB27860 SGB40294 (Muribaculaceae)* (0.16% ± 0.06 vs. 0, *p* = 5.19E-03), *GGB24148 SGB35952 (Muribaculaceae)* (0.78% ± 0.25 vs. 0, *p* = 4.81E-03), *GGB46163 SGB63931 (Bacteroidota)* (0.83% ± 0.22 vs. 0.07% ± 0.06 *p* = 6.82E-03), *Barnesiella* sp. *WM24* (6.95% ± 1.61 vs. 2.01% ± 1.06, *p* = 0.02), *GGB27914 SGB40352 (Muribaculaceae)* (0.16% ± 0.07 vs. 0.01% ± 0.01, *p* = 0.02), *GGB30454 SGB43516 (Oscillospiraceae)* (0.21% ± 0.07 vs. 0, *p* = 0.02), *GGB24127 SGB35930 (Muribaculaceae)* (0.15% ± 0.07 vs. 8.95E-03% ± 8.95E-03, *p* = 0.02), *Bacteroidales bacterium* (18.81% ± 5.04 vs. 5.53% ± 2.44, *p* = 0.03), *Duncaniella dubosii* (4.73% ± 1.08 vs. 1.64% ± 0.75, *p* = 0.03), *GGB27782 SGB40196 (Rikenellaceae)* (0.48% ± 0.24 vs. 0.05% ± 0.05, *p* = 0.04), *GGB30464 SGB43538 (Oscillospiraceae)* (0.06% ± 0.03 vs. 0, *p* = 0.04), *Muribaculaceae bacterium isolate 013 NCI* (0.07% ± 0.04 vs. 0, *p* = 0.03), *GGB27929 SGB40368 (Bacteroidota)* (0.25% ± 0.06 vs. 0.11% ± 0.09, *p* = 0.04), *GGB27873 SGB40307 (Muribaculaceae)* (0.08% ± 0.04 vs. 0, *p* = 0.04) compared to the LPS-treated group.

On the contrary, in the “LPS group,” compared to the control group, a higher number of representatives of the genus *Oscillospiraceae bacterium* (0.67% ± 0.19 vs. 0.13% ± 0.11, *p* = 0.04) was found.

**Figure 7:**
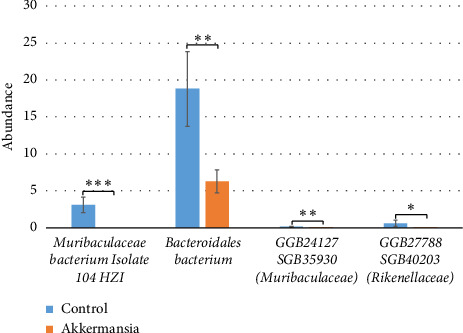
Statistically significant differences between the fecal microbiota of the “control group” and the “Akkermansia group” ^∗^*p* ≤ 0.05, ^∗∗^*p* ≤ 0.01, and ^∗∗∗^*p* ≤ 0.001.

There were also statistically significant differences at the species level between the “control group” and the “Akkermansia group” (Figure 7). We observed an increase in the abundance of the *Muribaculaceae bacterium isolate 104 HZI* (3.13% ± 1.06 vs. 0, *p* = 7.18E-05), *Bacteroidales bacterium* (18.81% ± 5.04 vs. 6.29% ± 1.55, *p* = 8.73E-03), *GGB24127 SGB35930 (Muribaculaceae)* (0.15% ± 0.07 vs. 2.80E-04% ± 2.80E-04, *p* = 6.60E-03), *GGB27788 SGB40203 (Rikenellaceae)* (0.63% ± 0.46 vs. 7.85E-03% ± 7.85E-03, *p* = 0.04).

**Figure 8:**
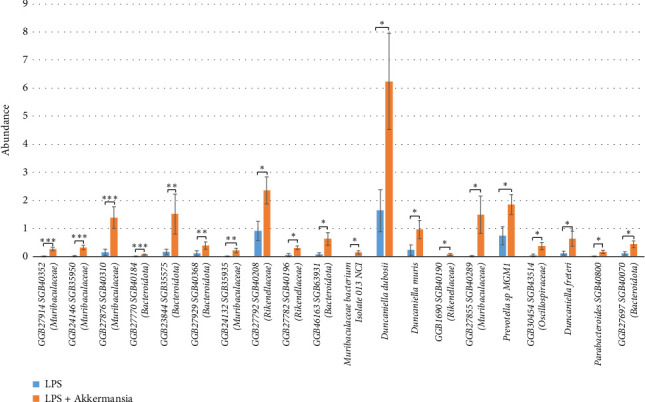
Statistically significant differences between the fecal microbiota of the “LPS group” and the “Akkermansia + LPS group.” ^∗^*p* ≤ 0.05, ^∗∗^*p* ≤ 0.01, and ^∗∗∗^*p* ≤ 0.001.

In the “Akkermansia + LPS group,” in comparison with the “control group,” we recorded a statistically significant differences of species *GGB27914 SGB40352 (Muribaculaceae)* (0.27% ± 0.06 vs. 0.01% ± 9.63E-03, *p* = 3.76E-05), *GGB24146 SGB35950 (Muribaculaceae)* (0.32% ± 0.08 vs. 0.02% ± 0.1, *p* = 1.08E-04), *GGB27876 SGB40310 (Muribaculaceae)* (1.38% ± 0.38 vs. 0.14% ± 0.11, *p* = 8.35E-04), *GGB27770 SGB40184 (Bacteroidota)* (0.07% ± 0.02 vs. 4.41E-03% ± 4.41E-03, *p* = 9.51E-04), *GGB23844 SGB35575 (Bacteroidota)* (1.51% ± 0.71 vs. 0.16% ± 0.09, *p* = 2.85E-03), *GGB27929 SGB40368 (Bacteroidota)* (0.39% ± 0.13 vs. 0.10% ± 0.09, *p* = 4.91E-03), *GGB24132 SGB35935 (Muribaculaceae)* (0.21% ± 0.07 vs. 2.68E-03% ± 2.68E-03, *p* = 5.43E-03), *GGB27792 SGB40208 (Rikenellaceae)* (2.35% ± 0.47 vs. 0.91% ± 0.33, *p* = 0.01), *GGB27782 SGB40196 (Rikenellaceae)* (0.30% ± 0.07 vs. 0.05% ± 0.05, *p* = 0.01), *GGB46163 SGB63931 (Bacteroidota)* (0.62% ± 0.23 vs. 0.08% ± 0.06, *p* = 0.01), *Muribaculaceae bacterium isolate 013 NCI* (0.15% ± 0.06 vs. 0, *p* = 0.02), *Duncaniella dubosii* (6.23% ± 1.72 vs. 1.64% ± 0.75, *p* = 0.02), *Duncaniella muris* (0.96% ± 0.32 vs. 0.22% ± 0.18, *p* = 0,02), *GGB1690 SGB40190 (Rikenellaceae)* (0.07% ± 0.04 vs. 0, *p* = 0.02), *GGB27855 SGB40289 (Muribaculaceae)* (1.49% ± 0.67 vs. 0.01% ± 0.01, *p* = 0.02), *Prevotella* sp. *MGM1* (1.85% ± 0.36 vs. 0.74% ± 0.33, *p* = 0.03), *GGB30454 SGB43514 (Oscillospiraceae)* (0.36% ± 0.13 vs. 0.04% ± 0.04, *p* = 0.03), *Duncaniella freteri* (0.63% ± 0.27 vs. 0.12% ± 0.07, *p* = 0.04), *Parabacteroides SGB40800* (0.16% ± 0.06 vs. 3.17E-03% ± 3.17E-03, *p* = 0.04), *GGB27697 SGB40070 (Bacteroidota)* (0.43% ± 0.13 vs. 0.11% ± 0.05, *p* = 0.04) (Figure 8).

**Figure 9:**
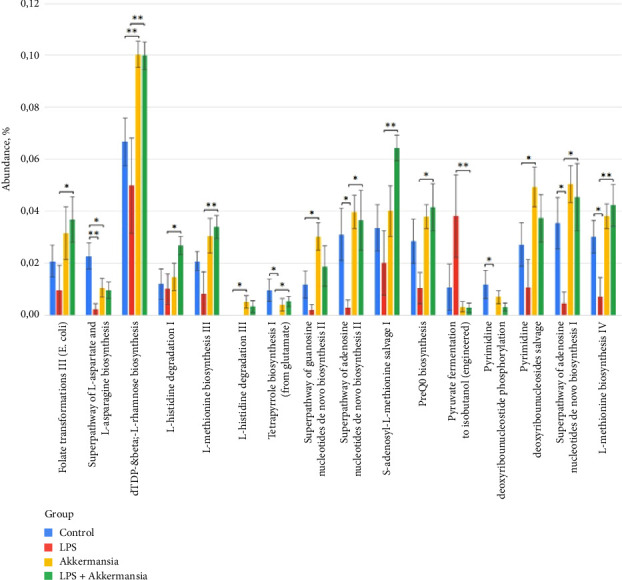
Differences in the prevalence of metabolic pathways between studied groups. ^∗^*p* ≤ 0.05 and ^∗∗^*p* ≤ 0.01.

There is a reliable reduction in the prevalence of metabolic pathways after LPS injections, compared with the control group: the superpathway of L-aspartate and L-asparagine biosynthesis (0.02 ± 4.99E-03 vs. 2.22E-03 ± 2.22E-03, *p* = 5.53E-03), the pathway of tetrapyrrol I biosynthesis (from glutamate) (9.41E-03 ± 4.34E-03 vs. 0, *p* = 0.04), the superpathway of the de novo II adenosine nucleotides biosynthesis (0.03 ± 0.01 vs. 2.86E-03 ± 2.86E-03, *p* = 0.02), the pyrimidine deoxyribonucleotide phosphorylation pathway (0.01 ± 5.36E-03 vs. 0, *p* = 0.04), superpathway of the de novo adenosine nucleotide biosynthesis I (0.04 ± 0.01 vs. 4.43E-03 ± 4.43E-03, *p* = 0.02), and IV pathway of L-methionine biosynthesis (0.03 ± 6.24E-03 vs. 7.13E-03 ± 7.13E-03, *p* = 0.02) (Figure 9).

There was also an inhibition of the activity of a variety of metabolic pathways in the group exposed to *A*. *muciniphila* compared to that of the control group: the superpathway of biosynthesis of L-aspartate and L-asparagine (0.02 ± 4.99E-03 vs. 0.01 ± 3.64E-03, *p* = 0.04), the biosynthesis pathway dTDP-beta-L-rhamnose (0.10 ± 5.04E-03 vs. 0.06 ± 0.01, *p* = 4.19E-03), L-histidine degradation pathway III (4.99E-03 ± 2.38E-03 vs. 0, *p* = 0.04), de novo guanosine nucleotide biosynthesis superpathway II (0.03 ± 5.23E-03 vs. 0.01 ± 5.16E-03, *p* = 0.02), and the disposal pathway of pyrimidine deoxyribonucleosides (0.04 ± 7.61E-03 vs. 0.03 ± 0.01, *p* = 0.04) (Figure 9).

On the contrary, when *A*. *muciniphila* and LPS injections were combined, the negative effects of LPS were leveled, compared with the group receiving only LPS injections: the folate III (E) conversion pathway (0.04 ± 8.71E-03 vs. 9.61E-03 ± 9.61E-03, *p* = 0.04), dTDP-beta-L-rhamnose biosynthesis pathway (0.09 ± 5.34E-03 vs. 0.05 ± 0.02, *p* = 4.62E-03), I-histidine degradation pathway (0.03 ± 3.48E-03 vs. 0.01 ± 5.94E-03, *p* = 0.01), L-methionine biosynthesis pathway (0.03 ± 4.37E-03 vs. 8.24E-03 ± 8.24E-03, *p* = 6.85E-03), tetrapyrrole biosynthesis pathway I (from glutamate) (5.24E-03 ± 1.91E-03 vs. 0, *p* = 0.04), the superpathway of adenosine nucleotides de novo II (0.04 ± 0.01 vs. 86E-03 ± 2.86E-03, *p* = 0.03), the rescue pathway of S-adenosyl-L-methionine I (0.06 ± 4.86E-03 vs. 0.02 ± 0.01, *p* = 1.64E-03), PreQ0 biosynthesis pathway (0.04 ± 0.01 vs. 0.01 ± 5.98E-03, *p* = 0.02), de novo adenosine nucleotide biosynthesis superpathway I (0.04 ± 0.01 vs. 4.43E-03 ± 4.43E-03, *p* = 0.02), and L-methionine biosynthesis pathway IV (0.04 ± 8.02E-03 vs. 7.13E-03 ± 7.13E-03, *p* = 8.49E-03). However, the pyruvate fermentation pathway to isobutanol (engineered) was more common in the “LPS group” (0.04 ± 0.02 vs. 2.89E-03 ± 1.87E-03, *p* = 7.63E-03) (Figure 9).

## 4. Discussion

The study revealed the effect of *A*. *muciniphila* consumption on the gut microbiome of mice against the background of induced systemic inflammation. Thus, changes in the relative abundance of some bacterial families were detected. The analysis of the results showed a statistically significant increase in the number of members of bacterial families: *Muribaculaceae, Oscillospiraceae, Rikenellaceae*. Representatives of the *Muribaculaceae* family (phylum Bacteroidota) are known to be key bacteria in the intestinal microbiota of mice and have also been identified in the intestines of other homeothermic animals [25]. In turn, this family is of interest due to its beneficial role in maintaining the health of the host. In addition, *Muribaculaceae* interacts well with probiotics, for example, *Bifidobacterium* and *Lactobacillus*; thus, the bacteria of this family may be potentially probiotic [26]. *Muribaculaceae* members are also known to ferment endogenous and exogenous polysaccharides to produce fatty acids, particularly propionate. These bacteria have also been reported to be able to break down oxalates in the organism [27]. It should be noted that *Muribaculaceae* are widely distributed in the intestines of mice. In turn, propionate is associated with intestinal health and an increase in life expectancy in mice [31]. It is also important to note that *Muribaculaceae* were also enriched in mice receiving fecal transplants from donor mice on a calorie-restricted diet, and metabolic improvements were noted in recipients as well [28]. In general, dietary changes negatively affect the relative abundance of *Muribaculaceae* [29].

In addition, the role of this bacterium in the course of metabolic diseases has been revealed. A study of a drug aimed at treating diabetes showed that members of the *Muribaculaceae* family were significantly more numerous in mice treated with the drug. These may indicate that members of the *Muribaculaceae* probably produce propionate and are numerous and diverse in the gut of mice [30]. Summarizing all the data, it can be concluded that members of the bacterial family *Muribaculaceae* in the future will probably be able to become immune bacteria in response to diseases. Thus, consumption of the bacterium can improve the immune system of mice through interaction with *Muribaculaceae* family members.

The effect of increasing the relative abundance of *Oscillospiraceae* bacteria in the intestines of mice after consumption of *A. muciniphila* may also indicate an improvement in the morphofunctional characteristics of mice. It has been established that bacteria of the *Oscillospiraceae* family are one of the common members of the intestinal microbiota associated with markers of health, such as microbial diversity and slimness, and are assumed to be capable of exerting an anti-inflammatory effect [31]. In addition, it is worth noting that numerous members of the *Oscillospiraceae* family exist in anaerobic environments, intestines and rumen, and play a vital role in the decomposition of carbohydrates and in the production of short- and medium-chain fatty acids, including butyrate, and may become a reference point for identifying “next-generation probiotics” [32, 33]. In addition, a number of studies have shown that this family is reduced in painful conditions, negatively correlates with the development of inflammatory diseases, and obesity; thus, the abundance of *Oscillospiraceae* is noted in the healthy control group [34]. The establishment of the fact of an increase in the relative content of *Oscillospiraceae* undoubtedly indicates positive processes in the gut of mice. In addition, it is worth mentioning that an analysis of the data we obtained showed that the representatives of *Oscillospiraceae* were significantly more abundant in the “control group” relative to the “LPS group.” These results may demonstrate the negative effects of LPS injections on the body of mice, which leads to the depletion of beneficial bacteria.

The third family *Rikenellaceae*, which showed an increase in its relative abundance in the gut of mice after consuming *A*. *muciniphila*, may also indicate a positive effect of this bacterium on the gut microbiome in conditions of systemic inflammation. It is known that members of the *Rikenellaceae* family belonging to the Bacteroidetes phylum are hydrogen-producing, which are able to protect body cells from damage from oxidative stress [35]. In addition, the number of representatives of this family can be increased by lactulose addition to mice [36, 37]. Members of the *Rikenellaceae* family are able to modulate obesity processes by producing acetate and propionate of short-chain fatty acids [38]. Previously, an increase in the number of the *Rikenellaceae* family in mice with diabetes was reported, which is associated with the pathological progression of inflammatory bowel disease [39]. It is also noted that this family outnumbers other common bacteria of the digestive tract when grown on mucin-rich media; in addition, representatives of the *Rikenellaceae* usually ferment carbohydrates or proteins [40, 41]. However, there is evidence that members of the *Rikenellaceae* family can be used as protective agents against cardiovascular and metabolic diseases associated with visceral fat, and thus they can serve as potential markers of healthy aging and, possibly, longevity [42]. It was also previously established that taking probiotics significantly increased the number of representatives of the *Rikenellaceae* family [43]. The results of our study showed a decrease in members of this bacterial family in the “LPS group” and “Akkermansia group” relative to the control group of mice. All this indicates the ambiguous role of *Rikenellaceae* family in the development of different illnesses and the formation of the gut microbiome and requires a more detailed study.

Interestingly, an increase in the above-described bacteria was previously detected under the action of inulin. Inulin in the intestine of mice can produce an increase in the number of *Muribaculaceae* (*Duncaniella muris*) and *Oscillospiraceae* [44].

Our exploration also noted that an increase in the relative diversity of three bacterial species (*Dunaliella muris*, *Dunaniella dubosii*, and *Dunaniella freteri*) was shown after consuming *A*. *muciniphila*. Noteworthy is the fact that currently, only three species are known for the genus *Dunaliella*: *D*. *muris*, *D*. *dubosii*, and *D*. *freteri*, which exhibit different abundance behaviors depending on gender, genotype, and age [45].

The bacteria *D*. *dubosii* play a key role in binding tryptophan supplementation to positive changes in the gut microbiome, immune modulation, and increased survival during brain tumor progression [46]. This bacterial species also shows high activity of β-glucosidase, α-arabinase, and α-fucosidase [26]. A study by Chen J. showed that the number of Duncaniella dubosii was lower in Atg5 knockout mice than in the control group, indicating the potential pathogenicity of these bacteria [47]. At the same time, *Duncaniella dubosii* is positively associated with the fecal microbial transfer procedure [48]. According to the results of our study, the abundance of this bacterial species in the control group was higher relative to the “LPS” group; in addition, an increase in the abundance of this species relative to the “LPS group” was recorded for the study “Akkermansia + LPS group.” These may indicate the beneficial functions of *Duncaniella dubosii*, which is confirmed by literature data.

The abundance of bacteria of the species *Dunaliella dubosii* and *Dunaniella muris* was also recorded for mice fed with extruded food containing dietary fiber [49].

According to the results of our study, it was shown that the number of *Duncaniella dubosii* bacteria in the “LPS group” decreased relative to the control group, while for the “Akkermansia + LPS group,” an increase in this bacterial species was shown in comparison with the “LPS” group. The abundance of bacterial species *Duncaniella muris* and *Duncaniella freteri* was also shown for the “Akkermansia + LPS group” relative to the “LPS group” of mice. These results may indicate a beneficial effect of the probiotic supplement on the inflammation induced by LPS injections.

The results of the study should be interpreted taking into account its limitations, primarily the small sample size. For further research on *Akkermansia muciniphila*, it is necessary to increase the number of individuals studied, as this is necessary to ensure a fair presentation of the results of statistical tests. Also, in future studies, it is necessary to conduct an analysis regarding several control points throughout the entire period of application of *Akkermansia muciniphila*.

During the study, it was noted that the probiotic supplement had a beneficial effect on metabolic pathways, the work of which was significantly disrupted by the action of LPS. However, the opposite was observed for the pyruvate-to-isobutanol fermentation pathway; this metabolic pathway was significantly increased relative to all the studied groups in the group of mice that were injected with LPS but not supplemented with *A. muciniphila*. Interestingly, this metabolic pathway, an engineering pathway not found in nature in any known organism, was constructed in a living cell using metabolic engineering [50].

## 5. Conclusion

According to the results of the study, representatives of the bacterial families *Muribaculaceae, Rikenellaceae,* and *Oscillospiraceae* showed a decrease in abundance under the influence of LPS; however, when consuming *A. muciniphila*, the number of family members increased. In addition, an assessment of the effect of LPS and *A. muciniphila* on metabolic pathways showed statistically significant differences for pathways of synthesis and degradation of amino acids; transform folic acid; synthesis of various sugars. In addition, the consumption of *A. muciniphila* increased the alpha diversity of bacteria in the gut (Shannon index) against the background of induced inflammation. This indicates that the bacterium *A*. *muciniphila* is able to modulate the gut microbiome in conditions of systemic inflammation.

It is noteworthy that *A*. *muciniphila* is capable of not only shifting the established microbiological balance in the intestine but also influencing the relative abundance of genes for various bacterial metabolic pathways. The results of our study on the effect of the bacterium *A. muciniphila* on the gut microbiome of mice against the background of induced systemic inflammation confirmed that a probiotic supplement based on this bacterial species is able to alleviate the negative effects of LPS injections on mice by correcting the gut microbiota. Thus, this bacterium can potentially be considered as an additional therapy for diseases accompanied by general inflammatory processes.

## Data Availability

The data that support the findings of this study are openly available in NCBI BioProject database at https://www.ncbi.nlm.nih.gov/bioproject/?term=PRJNA1230546.
